# Advancing Alzheimer's Diagnosis with AI-Enhanced MRI: A Review of Challenges and Implications

**DOI:** 10.2174/011570159X353595250303064846

**Published:** 2025-07-30

**Authors:** Zahra Batool, ShanShan Hu, Mohammad Amjad Kamal, Nigel H. Greig, Bairong Shen

**Affiliations:** 1 Center for High Altitude Medicine, Institutes for Systems Genetics, Frontiers Science Center for Disease-related Molecular Network, West China Hospital, Sichuan University, Chengdu, Sichuan, 610041, China;; 2 King Fahd Medical Research Center, King Abdulaziz University, Jeddah, 21589, Saudi Arabia;; 3 Department of Pharmacy, Faculty of Health and Life Sciences, Daffodil International University, Birulia, Savar, Dhaka -1216, Bangladesh;; 4 Centre for Global Health Research, Saveetha Medical College and Hospital, Saveetha Institute of Medical and Technical Sciences, Chennai, Tamil Nadu, India;; 5 Enzymoics, Hebersham, NSW 2770, Novel Global Community Educational Foundation, Australia;; 6 Drug Design & Development Section, Translational Gerontology Branch, National Institute on Aging, National Institutes of Health, Bethesda, Maryland, 21224, USA

**Keywords:** Artificial Intelligence, Alzheimer's disease, neurodegeneration, magnetic resonance imaging, deep learning models, convolution neural networks (CNNs), impaired cognition

## Abstract

Neurological disorders are marked by neurodegeneration, leading to impaired cognition, psychosis, and mood alterations. These symptoms are typically associated with functional changes in both emotional and cognitive processes, which are often correlated with anatomical variations in the brain. Hence, brain structural magnetic resonance imaging (MRI) data have become a critical focus in research, particularly for predictive modeling. The involvement of large MRI data consortia, such as the Alzheimer's Disease Neuroimaging Initiative (ADNI), has facilitated numerous MRI-based classification studies utilizing advanced artificial intelligence models. Among these, convolutional neural networks (CNNs) and non-convolutional artificial neural networks (NC-ANNs) have been prominently employed for brain image processing tasks. These deep learning models have shown significant promise in enhancing the predictive performance for the diagnosis of neurological disorders, with a particular emphasis on Alzheimer's disease (AD). This review aimed to provide a comprehensive summary of these deep learning studies, critically evaluating their methodologies and outcomes. By categorizing the studies into various sub-fields, we aimed to highlight the strengths and limitations of using MRI-based deep learning approaches for diagnosing brain disorders. Furthermore, we discussed the potential implications of these advancements in clinical practice, considering the challenges and future directions for improving diagnostic accuracy and patient outcomes. Through this detailed analysis, we seek to contribute to the ongoing efforts in harnessing AI for better understanding and management of AD.

## 1 INTRODUCTION

Alzheimer's disease (AD) is a common neurodegenerative disorder [1, 2], affecting 50 million world population [3, 4]. In 2019, 55 million people were estimated to have dementia across the world, and this is predicted to increase to 139 million by 2050, according to the WHO [5-7]. Meanwhile, the annual cost of dementia has been estimated to stand at US $1.3 trillion in 2019, a figure set to more than double by 2030 to $2.8 trillion. AD is caused by several symptoms, such as dementia, mood alterations, disorientation, and aphasia [8]. AD has been reported as a result of the linkage between nerve cells, neurotransmitters, immune cells, and endocrine cells in several experimental studies [9-11].

Moreover, extracellular amyloid plaques are the causative agents for AD symptoms, which are mainly formed in the brain, with an arrangement of dystrophic neuritis and intra-neuronal neurofibrillary tangles [2, 12]. Furthermore, molecular mechanisms underlying AD pathology are relatively complex and are accompanied by key events leading to the formation of these amyloid plaques. These plaques are further clustered into amyloid plaques or senile plaques on blood vessels and also on the outside surface area of neurons. In addition to this, it leads to an intra-cellular aggregation of microtubule-associated Tau proteins as neurofibrillary tangles. Amyloid-beta (Aβ) is termed a major component resulting from amyloid precursor protein (APP), being present inside the brain and several peripheral tissues [12]. Hence, Aβ formation and its presence, along with plaques in cortical areas of the brain, could be a hallmark of AD pathology.

In addition to this, glymphatic dysfunction is increasingly recognized as a contributing factor in AD due to its role in clearing neurotoxic waste from the brain. The glymphatic system, which primarily functions during sleep, removes metabolic byproducts and proteins like amyloid-beta and tau, which are hallmarks of AD. In Alzheimer's, this waste clearance system becomes impaired, leading to the accumulation of these proteins, which are toxic to neurons and contribute to the formation of plaques and tangles associated with cognitive decline. A key element in glymphatic dysfunction in AD is the impairment of aquaporin-4 (AQP4) channels on astrocytic end-feet surrounding blood vessels, which normally facilitate cerebrospinal fluid (CSF) flow through brain tissue. Reduced AQP4 polarization impairs CSF–interstitial fluid exchange, diminishing the brain's ability to clear amyloid-beta and other wastes, creating a cycle of protein accumulation and neurodegeneration [13].

**Fig. (1):**
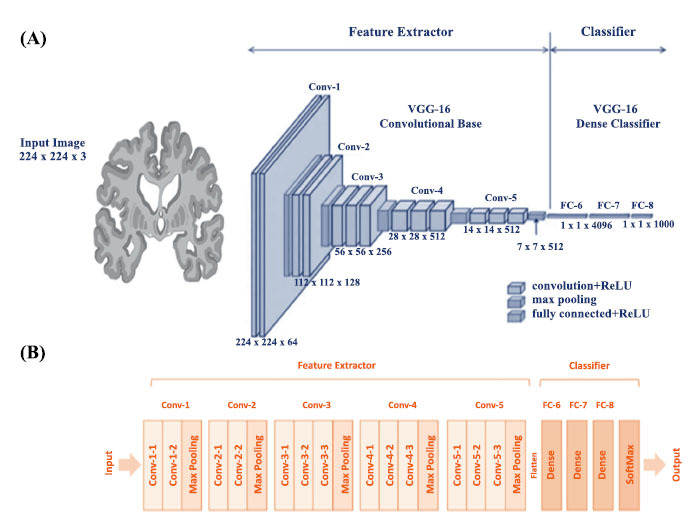
Convolution Neural Network working. The figure illustrates the working of convolutional neural network (CNN) used for image classification. (**A**) It starts with an input image of size 224×224×3224 \times 224 \times 3224×224×3 representing an RGB image. The network is divided into two main parts: a feature extractor and a classifier. (**B**) The feature extractor consists of five convolutional blocks (Conv-1 to Conv-5), where each block contains multiple convolutional layers followed by max-pooling layers to reduce spatial dimensions while increasing the depth of feature maps, capturing important patterns. After feature extraction, the classifier flattens these feature maps and passes them through three fully connected (FC) layers (FC-6, FC-7, and FC-8). FC-6 and FC-7 have 4096 neurons each, while FC-8, the final layer, outputs a probability distribution across 1000 classes, representing different image categories. The classifier concludes with a softmax layer that produces the final class predictions.

Meanwhile, the brain of AD patients undergoes an extent of oxidative damage caused by oxidative stress linked with an abnormal Aβ accumulation and deposition of neurofibrillary tangles [14] (Fig. **1**). Since oxidative stress has been suggested as another hallmark in AD progression in the brain [15], it occurs in the initial stages of AD in relation to the existence of Aβ [16]. Elevated levels of Aβ_1-40_ and Aβ_1-42_ are reported to be linked with an increased level of oxidation products from lipids, nucleic acids, and proteins in AD patient's cortex and hippocampus [16, 17]. Contrary to this, regions of the brain (*i.e*., cerebellum) possessing lower Aβ levels did not show higher concentrations of oxidative stress markers [18]. Furthermore, it is also assured that lipids and protein oxidation were being observed in brain regions richer with Aβ, where oxidized proteins could be identified by redox proteomics at the early stages of the disease [15]. Additionally, apart from the production of reactive oxygen species by Aβ peptides in the presence of metal ions, mitochondrial dysfunction is also involved in AD pathology through mitochondrial reactive oxygen species generation [19].

Neuroimaging techniques, particularly magnetic resonance imaging (MRI), provide crucial insights into real-time structural changes occurring within the brain, especially in the context of AD pathology [20]. MRI has emerged as a powerful tool for assessing medial temporal lobe atrophy, which is a key indicator of AD [21]. This atrophy is closely associated with the neurodegenerative process characteristic of AD, making MRI-based assessments vital for accurate clinical diagnosis. In addition to its diagnostic capabilities, MRI served as a non-invasive method to monitor the longitudinal progression of both cortical grey and white matter [22]. By allowing researchers and clinicians to visualize changes over time, MRI facilitated a deeper understanding of how AD evolved, enabling more personalized approaches to patient care.

Moreover, MRI can identify additional abnormalities in the brains of patients diagnosed with AD, such as issues related to small vessels [20]. These findings are critical, as they can inform us about coexisting vascular conditions that may complicate AD and affect overall cognitive function [21-23]. Overall, these investigations underscored the pivotal role of MRI in not only the early detection of AD but also in tracking disease progression and elucidating the structural transformations that accompany this complex neurodegenerative disorder. By enhancing our understanding of these changes, MRI contributed significantly to the development of more effective diagnostic and therapeutic strategies for AD [23, 24].

Meanwhile, various multicenter initiatives, such as the Alzheimer’s Disease Neuroimaging Initiative (ADNI), have been instrumental in collecting extensive datasets. These programs have amassed rich MRI data alongside other crucial information, such as clinical assessments and cognitive evaluations [25, 26]. Leveraging advanced artificial intelligence (AI) technologies, including machine learning and deep learning, researchers are analyzing these comprehensive datasets to enhance the diagnostic capabilities of automated systems. By integrating AI with large-scale neuroimaging data, these initiatives aimed to refine algorithms, improve their predictive power, and ultimately facilitate more effective clinical applications for diagnosing and monitoring AD [27].

Despite the growing interest and research surrounding various AI methodologies, there remains a significant lack of comprehensive evaluations regarding the features and efficacy of these technologies, particularly in the context of MRI for AD pathology [28]. This gap highlighted the need for a systematic synthesis of contemporary literature, which is essential for understanding the current state of research, emerging perspectives, and the obstacles that hinder advancements in MRI technology related to AI applications for AD diagnosis [29, 30]. This situation underscored the urgent need to advance research in this critical field.

Therefore, the objective of this paper was to review different challenges associated with AD diagnosis by MRI and the assistance of AI to cope with these problems. In this regard, particular emphasis was given to the roles of convolutional neural networks (CNNs) and non-convolutional artificial neural networks (NC-ANNs) in AD diagnosis by MRI. By addressing these objectives, this paper aims to critically evaluate the current capabilities and limitations of AI-driven MRI approaches in the context of AD diagnosis.

## 2 SEARCH STRATEGY

In this review, two independent researchers performed a literature search for AD, AI, and MRI in the electronic databases of PubMed, Web of Science, Google Scholar, and Cochrane (CENTRAL) to obtain published articles on AI-enhanced MRI diagnosis and their association with AD diagnosis. We used the following keywords and various combinations of the keywords or similar words (*e.g*., MeSH terms in PubMed) for the search: “Alzheimer’s Disease” OR “Neurogenerative Disorder” OR “Cognitive Disorder” AND “AI” OR “Artificial Intelligence” AND “Convolutional Neural Network” OR “CNN” OR “1D CNN” OR “2 D CNN” OR “3D CNN” AND “Non-Convolutional Artificial Neural Network” OR “NC-ANNS” AND “Magnetic Resonance Imaging-Convolutional Neural Network” OR “MRI” OR “MRI-CNN” OR “Magnetic Resonance Imaging-Artificial Neural Network” *etc*.

### 2.1 Inclusion Criteria

This review considered all articles that specifically examined the use of AI algorithms to support MRI-based diagnosis of Alzheimer’s Disease (AD). To capture a comprehensive view of AI-assisted approaches, studies involving both Convolutional Neural Networks (CNN) and Non-Convolutional Artificial Neural Networks (NC-ANN) were included. These AI methodologies offer different approaches to analyzing MRI data; CNNs are particularly effective for image analysis, while NC-ANNs can provide alternative analytical perspectives. By including studies using both types of algorithms, this review aimed to provide a thorough assessment of how AI techniques are enhancing MRI diagnostics in identifying and evaluating AD.

### 2.2 Exclusion Criteria

Articles that did not explicitly focus on diagnosing Alzheimer’s Disease (AD) through MRI in combination with AI algorithms were excluded from this review. Specifically, studies that lacked a direct examination of MRI-based AD diagnosis using AI-driven methods—whether through CNN or NC-ANN—were not considered. This exclusion criterion ensured that the review remained tightly focused on research directly relevant to AI-enhanced MRI diagnostic approaches for AD, providing a comprehensive analysis of studies within this specific intersection of AI, MRI, and AD diagnosis.

## 3 COPING CHALLENGES ASSOCIATED WITH AD DIAGNOSIS BY MRI *VIA* AI MODELS

Evaluating Alzheimer’s Disease (AD) through magnetic resonance imaging (MRI) poses substantial challenges, primarily due to its reliance on the expertise of clinical professionals [31]. Interpreting MRI scans requires nuanced assessments of complex neuroanatomical changes associated with AD, such as medial temporal lobe atrophy and white matter lesions. These changes can be subtle and vary significantly among patients, complicating the diagnostic process. To aid clinicians, various rating scales have been developed to provide structured frameworks for assessing brain changes over time [23]. However, while these scales aim to standardize assessments, they are still subject to individual judgment and experience, which can introduce variability in diagnostic outcomes [32]. This subjectivity raises concerns about consistency, especially across diverse clinical settings.

One of the challenges in studying AD over time is the shift in diagnostic criteria, which affects how cases are confirmed and evaluated. Initially, AD diagnosis primarily relied on cognitive assessments and clinical evaluations, often confirmed only through post-mortem examination. However, as medical science has advanced, new diagnostic tools have emerged, such as Positron Emission Tomography (PET) scans and blood biomarkers. PET scans, for instance, can now detect amyloid plaques and tau proteins, which are hallmarks of AD, providing more precise in-life diagnosis. Similarly, blood biomarkers have become increasingly effective in detecting early signs of AD, even before symptoms appear. This shift in criteria presents a challenge: how can researchers reconcile imaging and diagnoses conducted in earlier times with those based on these newer techniques? Older studies and records lack this advanced data, making it difficult to compare findings over time. For example, a patient diagnosed with AD twenty years ago might not have undergone PET imaging or blood tests, potentially leading to less specific diagnoses than today’s methods allow.

In response to these challenges, advancements in computer technology have led to the development of automated MRI assessment software. These tools streamline the analysis process by using sophisticated algorithms and machine-learning techniques to segment and extract critical information from MRI scans [32-34]. By reducing reliance on human interpretation, they aim to enhance diagnostic accuracy and efficiency, potentially minimizing the subjective biases that can impact clinical decision-making. The implications of these automated systems are significant [35]. By improving objectivity in MRI assessments, they help ensure that patients receive timely and appropriate care, especially in the early stages of AD when intervention may be most beneficial [32]. Additionally, automated MRI assessment facilitates large-scale studies, enabling researchers to explore AD progression and responses to treatment more effectively [36].

However, translating these technological innovations into clinical practice has been fraught with barriers. One major challenge is proving the adaptability of automated approaches across various neurodegenerative diseases. For example, conditions like frontotemporal dementia and vascular dementia can resemble Alzheimer’s Disease (AD), making it essential that automated tools are validated for a broad spectrum of disorders to avoid misdiagnosis [34, 37]. This adaptability is crucial not only for broader applicability but also for building clinician confidence in AI tools. Additionally, AI algorithms are now being used to detect genetic and proteomic biomarkers, enabling earlier interventions. Cognitive and behavioral assessments have also advanced through AI, with tools that improve the accuracy of neuropsychological tests and analyze speech and language patterns for early dementia indicators [38]. Moreover, the reliability of diagnostic outcomes generated by these automated systems is paramount. Clinicians must be confident that the software can consistently deliver accurate results across diverse patient populations and imaging protocols [38]. This requires extensive validation studies, which are often resource-intensive and time-consuming. Without robust evidence supporting the reliability of these systems, there remains a significant barrier to their widespread adoption in clinical settings.

Additionally, there is an inherent “black box” nature to many AI models, which can lead to scepticism among healthcare professionals. The lack of transparency in how these algorithms make decisions can hinder trust, particularly in high-stakes scenarios like AD diagnosis, where treatment decisions hinge on accurate assessments. [39]. Consequently, there is a growing demand for explainable AI, which aims to provide insights into the decision-making processes of these models, thereby enhancing their interpretability and fostering greater trust among clinicians for AD diagnosis by MRI. Therefore, the SECNN-RF framework (Spectral Enhanced Convolutional Neural Network and Random Forest) has emerged as an advanced model that combines deep learning and traditional machine learning techniques for enhanced performance in tasks such as image classification, particularly in medical imaging and remote sensing. Incorporating explainable AI (XAI) principles into this framework enhances the interpretability of its predictions [40].

In conclusion, while automated MRI assessment tools hold the potential to revolutionize the diagnosis and monitoring of Alzheimer’s Disease (AD), several critical challenges remain. It is essential to address issues related to data variability, adaptability across various neurodegenerative diseases, reliability, and interpretability to ensure the successful integration of these technologies into clinical practice. Ongoing collaboration among AI researchers, clinicians, and data scientists will be vital for navigating these challenges and fully harnessing the potential of AI models, particularly Convolutional Neural Networks (CNNs) and Non-Convolutional Artificial Neural Networks (NC-ANNs), in enhancing the diagnosis and management of AD.

## 4 CONVOLUTION NEURAL NETWORKS (CNNS)

Convolutional Neural Networks (CNNs) are a class of artificial neural networks specifically designed for processing structured data, such as images [41, 42] (Fig. **1**). They excel at identifying critical features in input data, which optimizes performance across various applications. In recent years, CNNs have emerged as powerful tools for analyzing MRI data, particularly in the context of Alzheimer’s Disease (AD) [43]. Their capability to learn hierarchical features from images enables them to deliver highly accurate outputs, significantly enhancing the diagnostic process [44]. Typically, CNNs employ stacked architectures that combine multiple machine-learning algorithms to improve effectiveness. This integration facilitates a more comprehensive analysis of MRI scans, ultimately aiding in the early detection and monitoring of AD pathology [45]. This section has discussed different CNN studies of AI models for the diagnosis of AD using MRI (Table **1**).

### 4.1 T1WI Imaging-based CNNs

T1-weighted imaging (T1WI) is a type of MRI technique that highlights differences in the longitudinal relaxation times (T1) of tissues [46]. In T1-weighted images, tissues with shorter T1 relaxation times appear brighter, while those with longer T1 relaxation times appear darker. As is common with deep learning methodologies, studies in this field typically utilize datasets comprising tens of thousands of cases. Much of the existing research has primarily focused on CNNs applied to T1WI. However, investigations combining T1WI with fluorodeoxyglucose positron emission tomography (FDG-PET) data have yielded notably varied results, with diagnostic accuracies ranging from 90% to 98% [46, 47].

One study introduced a CNN classifier that integrated multimodal data from the hippocampal region, utilizing both T1WI and FDG-PET. This approach achieved an impressive accuracy of 90% in distinguishing Alzheimer’s Disease (AD) from healthy controls (HC) and 77% in differentiating HC from individuals with progressive mild cognitive impairment (MCI). Additionally, the integration of T2WI with CNNs has been shown to achieve a remarkable classification accuracy of 99.2% in distinguishing AD from HC [48].

Several other studies have explored the concept of brain age, employing CNNs to automatically extract signatures from T1WI data. These studies have investigated correlations between brain age and various factors, including cognitive performance, chronological age, health outcomes, and genetic data [49, 50]. For example, one study utilized 3D ResNet CNN modeling to ascertain brain age and evaluate the relationship between chronological age and predicted brain age, alongside assessing the neuropsychological performance of participants [49]. Conversely, another study employed DeepBrainNet, an advanced CNN model leveraging transfer learning capabilities, to classify neurological disorders based on MRI-derived brain age. Their findings indicated an accuracy of 86% in distinguishing AD from HCs and 70% in differentiating MCI from HC. These results underscore the effectiveness of using brain age as a biomarker and highlight the potential of advanced CNN architectures in enhancing diagnostic precision.

**Fig. (2):**
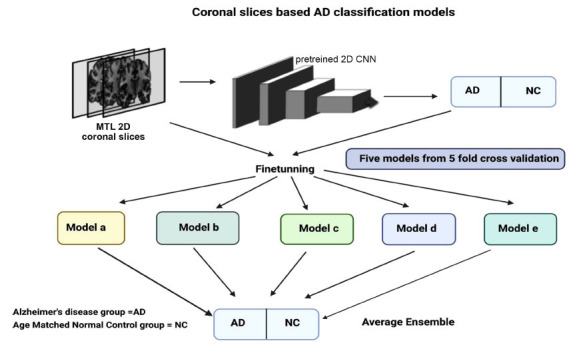
CNN coronal slices of T1-weighted images based AD classification models. This diagram illustrates method for classifying Alzheimer’s Disease (AD) using coronal slices from MRI scans. The process begins with the extraction of medial temporal lobe (MTL) 2D coronal slices from MRI images, which are then input into a pretrained 2D convolutional neural network (CNN) to extract relevant features. The dataset is split into five subsets for five-fold cross-validation, and five different models (Model a to Model e) are trained independently on each fold, with fine-tuning applied to each model. Each model then classifies the input as either AD (Alzheimer’s Disease) or NC (Age Matched Normal Controls). Finally, an average ensemble method combines the predictions of all five models to improve classification accuracy and reliability.

Additionally, a study developed CNN-based algorithms for classifying AD using MRI scans from AD patients and age- and gender-matched control subjects while considering variations in ethnicity and education level. The data were sourced from Seoul National University Bundang Hospital (SNUBH) and the Alzheimer's Disease Neuroimaging Initiative (ADNI) datasets. The authors trained their CNNs on five subsets of each population, utilizing coronal slices of T1-weighted images that specifically targeted the medial temporal lobes (Fig. **2**). This methodological choice is significant, as the medial temporal lobes are critical regions for understanding AD progression. To assess the robustness of their models, Bae *et al*. performed both within-dataset validation (using subsets from the same population) and between-dataset validation (using data from different populations). Their models achieved an area under the curve (AUC) ranging from 0.91 to 0.94 for within-dataset validation and 0.88 to 0.89 for between-dataset validation. These results highlight the models' potential for generalization across diverse patient populations with varying ethnic backgrounds and education levels.

### 4.2 Structure-focused Based CNN

The Structure-focused Neurodegeneration Convolutional Neural Network (SNeurodCNN) is a specialized deep learning architecture designed to analyze and predict neurodegenerative diseases by concentrating on structural features within brain imaging data. Recently, a novel SNeurodCNN was introduced [51, 52]. This innovative framework incorporates an image brightness enhancement preprocessor that utilizes gamma correction, significantly improving the quality of input images for more accurate analyses.

The primary function of SNeurodCNN is to analyze features such as focal structural atrophy, which are crucial for segmenting brain structures captured through MRI scans. The architecture consists of several key components, including two down-sampling convolutional blocks and two fully connected layers. These elements work together to enable the model to perform classification tasks effectively. Regularization techniques were also employed to optimize the learnable parameters and reduce the risk of overfitting.

By leveraging both mild sagittal and para-sagittal viewpoints from the Alzheimer's Disease Neuroimaging Initiative (ADNI) datasets, the model demonstrated impressive performance metrics. For the para-sagittal viewpoint, it achieved an accuracy of 97.8%, specificity of 97.0%, and sensitivity of 98.5%. Notably, the mild sagittal viewpoint provided even deeper insights, yielding an accuracy of 98.1%, specificity of 97.2%, and sensitivity of 99.0%.

While these results are commendable and suggest the potential of SNeurodCNN to enhance the classification of neurodegenerative changes, it is crucial to critically assess the model's generalizability across diverse populations and varying imaging conditions. Future research should focus on validating these findings in larger, more heterogeneous cohorts to ensure that the model's efficacy translates effectively into clinical practice.

### 4.3 Layer based CNN

A layer-based convolutional neural network (CNN) refers to an architecture built on a sequence of layers, each designed to perform a specific operation on the input data [53, 54]. These layers collaborate to learn hierarchical representations of the input, typically images, by gradually extracting more complex and abstract features as the data progresses through the network. For instance, one study explored various pooling functions and activation techniques using an eight-layer CNN to distinguish brain regions of healthy controls (HC) and Alzheimer’s Disease (AD) subjects. This research reported specificity and accuracy exceeding 97% in classifying AD *versus* HC [53].

Building on this work, another study replicated and expanded these findings by integrating CNNs with ensemble learning techniques. This approach focused on identifying discriminative brain regions to classify mild cognitive impairment (MCI) and AD pathology, achieving an impressive accuracy of 98% with an eight-layer CNN [54].

In a noteworthy advancement, a separate study developed a complex 12-layer CNN model, reporting both sensitivity and accuracy in its findings. Remarkably, this model achieved 99% accuracy for classifying AD *versus* healthy controls, utilizing separate training and validation datasets. This demonstrates the potential of deep learning techniques to significantly enhance diagnostic precision for AD [55].

### 4.4 1D-based CNN

A one-dimensional convolutional neural network (1D CNN) is specifically designed to process and analyze sequential data, such as time series, audio signals, or textual information [56]. Research studies have leveraged the strengths of both 1D CNNs and feedforward neural networks (FFNNs) to effectively extract features from raw data and perform high-level classification or regression tasks. For instance, one study employed 1D CNNs in conjunction with FFNNs for multi-class classification. This model integrated various data types, including MRI features, cerebrospinal fluid (CSF) biomarkers, ApoE4 genetic information, and cognitive test results.

This comprehensive approach demonstrated robust performance in differentiating between clinical diagnoses, with both algorithms achieving similar accuracies: 88% for the CNN model and 87% for the FFNN model. These findings underscore the effectiveness of utilizing diverse imaging modalities and data types to enhance the diagnostic accuracy of AD.

### 4.5 2D and 3D based CNN

Two-dimensional convolutional networks (2D CNNs) are specifically designed to process two-dimensional data, such as images. These networks perform convolutional operations over 2D spatial dimensions, making them suitable for tasks like image recognition, classification, object detection, and segmentation. In contrast, three-dimensional convolutional networks (3D CNNs) extend this concept to three-dimensional data [57]. By applying convolutional operations over 3D spatial dimensions, 3D CNNs are particularly effective for tasks involving volumetric data, such as video analysis and 3D medical imaging [58].

Several studies have explored the use of both 2D and 3D CNNs for Alzheimer’s Disease (AD) diagnosis. For instance, one study [59] proposed an innovative classification method that integrated multi-view 2D and 3D convolutions for MRI-based AD diagnosis. The authors first employed several sub-networks to extract local slice-level features from each MRI slice across various dimensions, allowing for a nuanced understanding of structural variations. Subsequently, a 3D CNN was utilized to capture global subject-level information from the MRI scans. By fusing both local and global data, the model aimed to identify the most discriminative features for AD classification. The results were promising, achieving accuracies of 90.2% on the ADNI-2 dataset and 85.2% on the ADNI-1 dataset, indicating the effectiveness of this multi-scale feature extraction method in enhancing AD diagnostic accuracy. However, further validation is necessary to assess the model’s robustness across diverse populations and imaging conditions.

Similarly, another study [57] introduced a model that integrated multi-view-slice attention with 2D and 3D CNNs. The authors focused on extracting local slice-level characteristics by utilizing multiple sub-networks, enabling detailed analysis of structural variations within individual slices. To enhance the model's effectiveness, they implemented a slice-level attention mechanism that emphasized specific 2D slices while filtering out redundant features. This optimization ensured that the most relevant information was considered during analysis. Following this, a 3D CNN captured global subject-level structural changes, providing a comprehensive understanding of the MRI data. The experiments conducted on a sample of 1,451 subjects from the ADNI-1 and ADNI-2 datasets yielded impressive results, achieving 91.1% accuracy for AD diagnosis and 80.1% for predicting mild cognitive impairment (MCI). These findings underscored the potential of this approach in improving diagnostic accuracy.

Moreover, another study [58] compared several deep learning models, including 2D and 3D CNNs and recurrent neural networks (RNNs), for AD classification using MRI scans. The authors initially split each MRI scan into 2D slices, which overlooked the spatial connections among these slices when using 2D CNNs on 3D MRI volumes. To address this limitation, they combined a CNNs with RNNs, enabling the 2D CNN + RNN model to better understand the relationships among 2D slices and their sequences. Notably, the feature extraction performed by the 2D CNN was independent of the RNN classification, leading the authors to replace the 2D CNNs with a 3D CNNs. This adjustment allowed for voxel-based decision-making, thereby leveraging the full volumetric information of the MRI data.

This study notably contributed to the field by introducing transfer learning from 2D image datasets to 3D CNNs. The results indicated that sequence-based decisions improved classification accuracy by 2% when distinguishing AD patients from healthy subjects. Furthermore, the 3D voxel-based method utilizing transfer learning outperformed other approaches, achieving diagnostic accuracy of 96.88%, 100% sensitivity, and 94.12% specificity. Overall, the integration of voxel-based methods with transfer learning from ImageNet to MRI datasets using 3D CNNs significantly enhanced classification outcomes compared to traditional methods.

### 4.6 Deep 3D-based CNNs

A deep 3D convolutional neural network (3D CNNs) is designed to process three-dimensional data, such as volumetric images or video data, capturing information across multiple spatial dimensions (x, y, and z). One study [60] proposed densely connected deep 3D CNNs that incorporated a connection-wise attention mechanism to effectively learn multi-level features from brain MRI images for Alzheimer’s Disease (AD) classification. The authors focused on extracting multi-scale features from pre-processed images, allowing for the integration of connections across different feature layers. This approach transformed the MRI data into compact, high-quality feature representations. To enhance the model’s ability to capture spatial information, the authors extended the convolution operation to deep 3D, enabling a more comprehensive analysis of the multimeric MRI data. This methodological innovation was significant, as it better reflected the complex anatomical structures associated with AD. The extracted features were integrated with those from all preceding layers, each weighted by different attention mechanisms, maximizing the model's discriminative power in classifying AD. The model was evaluated on a dataset comprising 968 subjects from the ADNI database, achieving an impressive accuracy of 97.35% for AD diagnosis. While these results are notable, further exploration of the implications of using attention mechanisms is needed to understand their contributions to feature selection and classification efficacy in practical applications.

Additionally, another study [61] developed a novel 3D deep CNN approach to differentiate accurately between AD, mild cognitive impairment (MCI), and healthy subjects using MRI images. The authors constructed a reference model based on the volumes and thicknesses of brain regions implicated in disease progression, which served as a benchmark for their deep learning model. Both models were evaluated on an internal held-out cohort from the ADNI and an external independent cohort from the National Alzheimer’s Coordinating Center (NACC). The deep learning model demonstrated promising performance, achieving an AUC of 85.12 and 89% accuracy in distinguishing between AD and MCI patients. While these results are encouraging, further investigation is warranted to assess the model's robustness across diverse populations and varying imaging conditions. Moreover, exploring the implications of volumetric and thickness metrics in the model could enhance our understanding of their contributions to accurate diagnosis and clinical relevance in AD research.

Recent evaluations indicate that many studies on AD have predominantly relied on single data modalities for predicting AD stages, a narrow approach that may overlook the disease's complex nature. In contrast, integrating multiple data modalities can provide a more nuanced understanding of AD progression. In this context, one study [62] adopted a multifaceted approach by leveraging deep 3D learning to analyze MRI data, single nucleotide polymorphisms (SNPs), and clinical assessments to classify patients into distinct categories: AD, MCI, and healthy controls (HC). The authors employed stacked denoising autoencoders to extract relevant features from clinical and genetic data, subsequently applying a 3D CNN for imaging analysis with 87% accuracy. While this method showcased innovative techniques, it raises questions about the potential limitations of feature extraction and the interpretability of deep learning models in clinical settings.

Another study [43] presented a novel deep 3D CNN architecture aimed at categorizing AD using MRI data from the ADNI datasets. This innovative approach employed two distinct CNN models, each featuring different filter sizes and pooling layers, which were concatenated at the classification layer. The proposed model demonstrated impressive performance metrics, achieving an accuracy of 99.43%, specificity of 99.57%, and sensitivity of 99.13%. While these results highlighted the network's capability to effectively capture and distinguish relevant features from MRI images, they also raised important questions regarding the generalization of such high performance across diverse clinical populations. The model's ability to classify various subtypes of AD and identify different stages was commendable; however, the clinical utility of these findings must be critically evaluated.

Meanwhile, another novel approach introduced by [46] employed a deep 3D capsule network along with a pre-trained 3D autoencoder. This innovative method achieved an impressive 94.6% accuracy in distinguishing between MCI/AD and 97.6% accuracy in classifying AD against healthy controls (AD/HC). These results indicate that this approach surpassed the performance of conventional PET or MRI data alone, highlighting the potential of integrating advanced deep learning techniques to improve diagnostic accuracy in AD.

### 4.7 3D Jacobian Domain-based CNN (JD-CNN)

A 3D Jacobian Domain-based Convolutional Neural Network (JD-CNN) is a specialized type of neural network that incorporates the concept of the Jacobian domain into its architecture [63]. The Jacobian matrix, which represents the derivatives of a vector-valued function, captures local variations and dependencies in three-dimensional data. One study [63] proposed a novel 3D JD-CNN for diagnosing Alzheimer’s Disease (AD), achieving impressive classification performance without relying on a landmark detection framework. The authors trained the JD-CNN model using specific features generated by transforming structural MRI (sMRI) data from the spatial domain into the Jacobian domain.

The JD-CNN was evaluated on baseline T1-weighted sMRI data collected from 154 healthy subjects and 84 AD patients from the ADNI datasets. The results indicated that this approach effectively circumvented challenges associated with landmark detection, potentially improving the reliability of AD diagnosis achieving an accuracy of 87.4%. While these findings are promising, further research is necessary to validate the JD-CNN's performance across diverse populations and imaging conditions. Additionally, exploring the clinical applicability of this model in real-world settings will be crucial for its integration into routine diagnostic practices.

In summary, the application of CNNs in diagnosing AD represents a significant advancement in neuroimaging analysis. Their ability to learn complex patterns and integrate multimodal data has enhanced diagnostic accuracy and facilitated early interventions [64]. The promising results from various studies underscore the potential of CNNs to revolutionize clinical practices in AD management. Future research should focus on refining these models and exploring their application in broader clinical settings to improve patient outcomes and deepen our understanding of AD progression.

## 5 NON-CONVOLUTIONAL ARTIFICIAL NEURAL NETWORKS (NC-ANNS)

A non-convolutional artificial neural network (NC-ANN) is a type of deep learning algorithm composed of interconnected layers of nodes known as artificial neurons. These networks enhance performance through self-tuning, enabling them to learn and adapt to complex data patterns [65]. NC-ANNs are frequently employed for analyzing large-scale datasets, such as those from the Alzheimer's Disease Neuroimaging Initiative (ADNI). This approach can be effectively compared with other algorithms for the early classification of Alzheimer’s Disease (AD).

Several studies have reported the high performance of NC-ANNs, largely due to advancements in technology [66-71]. This section will discuss the involvement of various NC-ANNs in the diagnosis of AD using MRI, as summarized in Table **2**.

### 5.1 Bi-directional Long Short-Term Memory (BiLSTM) based NC-ANNs

A Bi-directional Long Short-Term Memory (BiLSTM) based neural network combined with non-convolutional artificial neural networks (NC-ANNs) integrates the strengths of both BiLSTM and convolutional neural networks (CNNs) for tasks involving sequential and spatial data processing. Recently, Matlani *et al*. [70] published a study focused on automated Alzheimer's Disease (AD) diagnosis using this hybrid approach.

The authors began with improved adaptive Wiener filtering (IAWF) to enhance image quality, followed by principal component analysis (PCA) to extract features through a normalized global image descriptor (PCA-NGIST), which eliminated the need for image segmentation. Significant features were refined using the improved wild horse optimization algorithm (IWHO). For disease classification, the authors employed a hybrid method that combined BiLSTM with NC-ANN. Implemented on the MATLAB platform, their approach achieved impressive accuracies of 99.22% for ADNI datasets and 98.96% for OASIS datasets.

This represents a significant advancement in AD diagnosis, leveraging sophisticated deep-learning techniques and innovative feature extraction methods without explicit segmentation. The high accuracies reported underscore the potential of such hybrid models in clinical settings. However, the reliance on specific datasets and the MATLAB platform necessitates consideration of generalizability across broader populations and validation in real-world clinical scenarios.

### 5.2 Deep Boltzmann Machine-based NC-ANNs

A Deep Boltzmann Machine (DBM) based Artificial Neural Network (ANN) is a generative model composed of multiple layers of stochastic, binary units. As a type of Boltzmann Machine, DBMs can learn internal representations of data and are utilized for unsupervised learning tasks. In a study by [72], an innovative approach was introduced to extract high-level latent and shared representations from neuroimaging modalities using deep learning. This study focused on the diagnostic classification of Alzheimer’s Disease (AD) compared to healthy controls (HCs) and the prediction of progression from mild cognitive impairment (MCI) to AD, employing a deep Boltzmann machine.

The study reported notable achievements, including accuracies of 95% for distinguishing AD patients from HCs and 76% for predicting the conversion of MCI to AD. These results underscore the potential of deep learning models, such as deep Boltzmann machines, to uncover complex patterns and associations within neuroimaging data.

However, several factors warrant critical evaluation. Firstly, while high accuracies were achieved, the generalizability of the study may be influenced by the specific characteristics and demographics of the datasets used. Additionally, the interpretability of features learned by deep learning models like Boltzmann machines remains a challenge, limiting their clinical applicability and broader adoption.

### 5.3 Resting-state Functional MRI (rs-fMRI) Connectivity Data based NC-ANN

Resting-state functional MRI (rs-fMRI) connectivity data artificial neural networks (ANNs) refer to the application of ANNs to analyze and interpret functional connectivity data obtained from resting-state fMRI scans. Previous studies utilized rs-fMRI connectivity data combined with advanced machine learning methods to distinguish individuals with Alzheimer’s Disease (AD) from healthy controls (HCs). Both studies demonstrated significant improvements in classification accuracy, highlighting the potential of rs-fMRI as a diagnostic tool.

Specifically, another study focused on leveraging functional connectivity patterns observed in rs-fMRI data using machine learning techniques to enhance diagnostic accuracy. Their findings indicated that analyzing intrinsic brain activity during rest provides valuable insights that can differentiate between AD and HC with an accuracy of 86%, which is crucial for early diagnosis and intervention.

Meanwhile, another study extended this work by not only utilizing rs-fMRI connectivity data but also incorporating an advanced machine learning approach known as extreme learning machine (ELM). This method, along with a sophisticated multivariate pattern analysis feature selection algorithm, reportedly achieved near-perfect classification performance with an accuracy of 86.8%. The use of ELM is noteworthy because it is designed to efficiently handle large datasets, offering faster training times and potentially better generalization compared to traditional neural networks for AD classification.

### 5.4 Salient Skeletal Model based NC-ANN

A Salient Skeletal Model-based Artificial Neural Network (ANN) is designed to process and analyze skeletal data, focusing on identifying and leveraging key or “salient” features of skeletal structures. Previous studies utilized this salient skeletal model to extract features from MRI scans, effectively identifying known brain networks and achieving high performance in classifying Alzheimer’s Disease (AD).

This research emphasized the potential of integrating various data modalities and advanced analytical techniques to enhance diagnostic accuracy in neurodegenerative disorders. By leveraging a skeletal model, the study highlighted significant structural elements in MRI data, facilitating the identification of distinct brain networks associated with AD. The salient skeletal model worked by emphasizing critical regions and connections within the brain that are most relevant for distinguishing AD from healthy controls (HCs).

By concentrating on these salient features, the model achieved an impressive accuracy of 95%, demonstrating robust classification results. The high performance reported in this study suggests that the skeletal model effectively captures the complex and subtle changes in brain structure indicative of AD. This approach contrasts with traditional methods that may rely on broader, less focused analyses of MRI data, which can be less sensitive to the specific alterations associated with AD.

### 5.5 Support Vector Machines (SVM) based NC-ANN

A Support Vector Machine (SVM) is a supervised machine learning algorithm primarily used for classification tasks. Both Support Vector Machines (SVM) and Artificial Neural Networks (ANN) are powerful techniques that can be combined to leverage their respective strengths. Recently, [71] conducted a study exploring MRI markers aimed at predicting Aβ-positivity in Alzheimer’s Disease (AD) pathology. They employed machine learning (ML) and SVM to analyze MRI data from 139 patients who underwent brain MRI and amyloid PET-CT scans. The study divided patients into Aβ-positive (Aβ[+]) and Aβ-negative groups, revealing significant differences in MRI markers between them.

The findings indicated that Aβ(+) individuals exhibited higher Fazekas scale scores for white matter hyperintensity (WMH) and cerebral microbleeds (CMB), reflecting more severe small vessel disease pathology. Additionally, Aβ(+) patients had larger third ventricle volumes, suggesting greater cerebral atrophy. An ML logistic regression model achieved an accuracy of 81.1% after incorporating regional brain volumes and the Mini-Mental State Examination (MMSE), demonstrating its potential to predict Aβ(+) status in AD pathology. This approach highlights the utility of advanced imaging techniques and computational methods in identifying subtle neuroimaging markers associated with AD.

Additionally, research by [76] documented a stacked network utilizing particle swarm optimization, which incorporated both NC-ANN and SVM algorithms for AD classification. This innovative approach enhanced classification accuracy to 89% by leveraging the strengths of multiple machine-learning techniques.

In summary, NC-ANNs have proven to be transformative tools in the early detection and classification of AD. Their capability to process and analyze large-scale neuroimaging datasets has significantly improved the accuracy and reliability of diagnostic assessments. The integration of various methodologies, such as hybrid models that combine NC-ANNs with Support Vector Machines and advanced feature selection techniques, has further optimized performance, allowing for more nuanced interpretations of complex data patterns. Moreover, the successful application of NC-ANNs in distinguishing between AD, Mild Cognitive Impairment (MCI), and healthy controls (HCs) underscores their potential to facilitate timely and effective clinical interventions. As research progresses, continued exploration of innovative approaches, including the incorporation of multimodal data from MRI and clinical assessments, will be crucial for refining these models.

## 6 CONCERNS REGARDING INTEGRATION OF MACHINE LEARNING (ML) ALGORITHMS FOR REAL-LIFE MEMORY CLINIC POPULATIONS

The integration of machine learning (ML) algorithms, particularly neural network approaches, into the diagnosis and management of Alzheimer’s Disease (AD) and related cognitive disorders in memory clinic populations raises several critical challenges. One major concern revolves around the inherent variability and uncertainty present in these patient populations. Unlike well-characterized datasets, such as those found in the Alzheimer’s Disease Neuroimaging Initiative (ADNI), which provides clear classifications of conditions based on standardized criteria, memory clinic patients often present a spectrum of cognitive impairments [10]. These impairments may not fit neatly into the established diagnostic categories of AD, mild cognitive impairment (MCI), or other neurodegenerative disorders. This complexity significantly complicates the training and validation of ML models, which thrive on well-defined patterns and labeled data.

In the context of neural network approaches, which are particularly powerful for pattern recognition in large datasets, the ambiguity associated with real-world memory clinic cases can pose substantial challenges. These algorithms typically rely on high-quality, labeled training data to learn to classify cases accurately [60]. However, when faced with patients who exhibit mixed symptoms or uncertain pathology—where the cognitive profile may overlap with both AD and other conditions—these models may struggle to generalize effectively. The nuanced presentations in clinical settings can lead to reduced classification performance, as neural networks may not have encountered similar cases during training. This limitation underscores the importance of developing models that can adapt to and learn from the variability present in real-world data rather than solely relying on well-defined training examples.

To enhance the diagnostic utility of ML algorithms in these complex clinical environments, there is an urgent need for researchers to interrogate a broader range of real-world databases. By expanding the scope of data analyzed, researchers can identify common patterns and features that extend beyond the well-characterized cases typically used in model training. Such efforts are essential to improve the generalizability of ML algorithms [69]. Moreover, they may reveal critical insights into the interactions between different cognitive impairments, potentially leading to a more comprehensive understanding of the underlying mechanisms of cognitive decline. Incorporating data from diverse sources not only enhances model robustness but also reflects the heterogeneity observed in clinical populations, thereby increasing the likelihood of effective implementation in everyday practice.

Additionally, the exploration of real-world databases can facilitate the development of innovative techniques for data augmentation and transfer learning, which could mitigate the challenges posed by small sample sizes in certain diagnostic categories. By utilizing existing datasets and applying advanced methodologies, researchers can effectively create more representative training sets that better capture the complexities of cognitive disorders. This approach could empower ML algorithms to learn from varied clinical presentations, thereby enhancing their predictive capabilities and improving diagnostic accuracy.

In conclusion, while ML algorithms possess the potential to transform the landscape of Alzheimer’s Disease diagnosis and management, their successful application in real-life memory clinic settings necessitates careful attention to the unique challenges posed by these populations. The development of adaptable models that can effectively handle ambiguous and heterogeneous cases, coupled with the interrogation of diverse datasets, is essential for ensuring that AI systems are reliably integrated into clinical practice. Such advancements will ultimately benefit patients, clinicians, and the broader healthcare system by facilitating timely and accurate diagnoses, enhancing patient care, and promoting a deeper understanding of the complex nature of cognitive decline.

## 7 IMPLICATION, ADVANTAGES AND LIMITATIONS OF NEURAL NETWORKS

This review emphasized the effectiveness of convolutional neural networks (CNNs) and non-convolutional artificial neural networks (NC-ANNs) in analyzing medical imaging data, particularly in the context of Alzheimer's Disease (AD). CNNs have demonstrated superior performance compared to other algorithms, establishing themselves as promising tools for various clinical applications [77]. Their ability to process complex imaging data makes them particularly useful for detecting patterns and abnormalities associated with AD. However, it is essential to recognize that each algorithm, including CNNs and NC-ANNs, has its strengths and weaknesses. For example, while CNNs excel at handling image data, NC-ANNs may be better suited for specific types of data or classification tasks. This variability underscores the need for careful selection of algorithms for brain MRI analysis, as the choice can significantly impact the understanding and diagnosis of AD [78].

Moreover, both CNNs and NC-ANNs are effective in solving regression and classification problems, which are vital for diagnosing and tracking the progression of AD. Despite their advantages, these networks require substantial computational resources and large datasets for training. The necessity for extensive data can limit their applicability, especially in settings where such datasets are not readily available. Additionally, while large T1-weighted MRI datasets are invaluable for training deep learning models, integrating these with other types of MRI scans can pose challenges [46, 47]. The diversity of imaging modalities may provide rich information, but insufficient data in some areas can impede the effective training of these complex models [48].

Another significant challenge is the “black box” nature of neural networks. The weights assigned to individual nodes within these networks are often difficult to interpret, making it challenging for researchers and clinicians to understand how decisions are made. This lack of transparency has spurred interest in developing explainable neural networks. These methods aim to enhance the interpretability of AI models, enabling users to gain insights into the decision-making processes of the algorithms [79]. Such advancements are crucial for building trust in AI applications in healthcare and ensuring that users can confidently apply these technologies in clinical settings.

Furthermore, the current understanding of AI in MRI analysis for AD remains limited. There is a pressing need to validate these AI systems across diverse populations and various imaging conditions to ensure their robustness and reliability. The integration of AI into clinical practice demands rigorous testing and clinical validation to ensure that diagnostic outcomes are both accurate and consistent.

Challenges such as potential biases in training datasets can lead to disparities in AI performance across different demographic groups, which may hinder generalizability. As AI technologies are increasingly implemented in real-world clinical scenarios, understanding their limitations becomes paramount. The risk of misdiagnosis or over-reliance on AI predictions could undermine the potential benefits of these advanced technologies. For instance, discrepancies in diagnostic criteria or variations in imaging protocols may affect the AI model's performance.

Therefore, ongoing research must address these challenges through comprehensive evaluation frameworks that incorporate diverse datasets and real-world conditions. Collaboration among AI developers, clinicians, and regulatory bodies will be essential to establish best practices for AI deployment in clinical settings. Ensuring transparent validation processes and establishing robust guidelines for clinical integration will help mitigate risks and enhance diagnostic accuracy and treatment outcomes for AD patients. Ultimately, the goal should be to create AI systems that not only support but also complement clinical expertise, leading to improved patient care and outcomes.

## CONCLUSION

Artificial intelligence (AI), particularly through machine learning and deep learning, has rapidly evolved to play a crucial role in facilitating MRI analysis. This evolution is significant for the early detection, diagnosis, and management of Alzheimer's Disease (AD). However, the effectiveness, transparency, and accuracy of these AI methods are essential for their successful implementation in clinical settings. The increasing accessibility of large datasets and the advancement of computing capabilities present promising opportunities for further AI development, especially within advanced deep learning techniques. These innovations have the potential to enhance our understanding of AD and improve its management, potentially revolutionizing clinical practice in the future.

Current AI literature has predominantly focused on the classification of AD and Mild Cognitive Impairment (MCI). Nevertheless, recent advancements in algorithms have also begun to incorporate automated brain age assessments. Accurately identifying subtle MRI features throughout the brain is crucial for the early detection of AD and the assessment of brain aging risks. To achieve this, more concerted efforts are necessary to explore the intricacies of brain imaging data. Future research should aim to develop innovative approaches that effectively capture the subtle changes in brain structure and function that are indicative of disease onset and progression. This will not only enhance early diagnostic capabilities but also support personalized treatment strategies that could significantly improve patient outcomes.

Looking ahead, it is important to consider whether well-trained AI models based on multimodal data, including various biomarkers in addition to MRI scans, will be able to diagnose AD based solely on MRI data or if a specific set of markers will be required. The integration of diverse data sources can enhance the diagnostic accuracy and robustness of AI systems, potentially allowing for more nuanced and reliable assessments of AD and its distinctions from other forms of dementia. We appreciate the importance of addressing differential diagnoses for other types of dementia. It is indeed crucial for clinicians to have a comprehensive understanding of the various neurodegenerative disorders, as this knowledge enables them to make more informed and accurate diagnoses. Incorporating a discussion on other dementias, such as vascular dementia and frontotemporal dementia, would provide valuable context. Each of these conditions presents distinct clinical features and underlying pathophysiological mechanisms that can overlap with or mimic AD. For instance, vascular dementia often arises from cerebrovascular disease and is characterized by sudden cognitive decline related to strokes, while frontotemporal dementia primarily affects younger individuals and involves changes in personality, behavior, and language. By briefly outlining the key clinical characteristics and diagnostic criteria for these dementias, we can equip clinicians with the necessary tools to differentiate between these disorders more effectively. Furthermore, understanding these distinctions can guide treatment strategies and management approaches, ultimately leading to better patient outcomes.

Finally, the relevance of these AI-driven approaches to treatment should be emphasized. Accurate diagnoses informed by advanced AI methodologies are crucial for developing personalized treatment plans tailored to individual patients. This can lead to more effective interventions and improved management of AD, ultimately enhancing the quality of life for patients and their families. By incorporating these considerations, the authors can provide a comprehensive outlook on the transformative potential of AI in the future landscape of Alzheimer's disease diagnosis and treatment.

## Figures and Tables

**Table 1:** Summary of all studies diagnosing AD by MRI using CNNs.

**Study Types**	**Aim of Study**	**Participants**	**Dataset**	**Strategy**	**Outcomes**	**Validation Approach**	**Performance (Accuracy %)**	**References**
T1WI Imaging based CNNs	To Integrate multimodal data from the hippocampal region for AD diagnosis	Healthy controls (HC) = 1506 and Alzheimer's disease (AD) = 1355 patients	Alzheimer's Disease Neuroimaging Initiative (ADNI)	Utilizing both T1-weighted imaging (T1WI) and fluorodeoxyglucose positron emission tomography (FDG-PET)	Effectively distinguished Alzheimer’s disease (AD) from healthy controls (HC)	K-Fold Cross-Validation	90%	[47]
T1WI Imaging based CNNs	To evaluate the relationship between chronological age and predicted brain age for AD diagnosis	Healthy controls (HC) = 1264 and Alzheimer's disease (AD) = 1888 patients	ADNI	Developed and employed 3D ResNet CNN modeling	Ascertained brain age and the relationship between chronological age and predicted brain age in AD subjects	Nested Cross-Validation	87%	[49]
T1WI Imaging based CNNs	To leverage the transfer learning capabilities for classifying AD diagnosis	Healthy controls (HC) = 1143, Mild cognitive impairment (MCI) = 2744 and Alzheimer's disease (AD) = 1045 patients	Local	Developed and employed DeepBrainNet model	Distinguished Alzheimer’s disease (AD) from healthy controls and mild cognitive impairment (MCI)	K-Fold Cross-Validation	86% for AD and 76% for MCI	[50]
T1WI Imaging based CNNs	To classify the AD patients and age-and gender-matched control subjects	Healthy controls (HC) = 987 and Alzheimer's disease (AD) = 1098 patients	ADNI	Developed and employed CNN-based algorithms	Obtained specific targeted medial temporal lobes to evaluate AD progression	Nested Cross-Validation	85 to 90%	[51]
Structure-focused based CNN	Predicted AD by focusing on structural features within brain imaging data	Healthy controls (HC) = 1934 and Alzheimer's disease (AD) = 1045 patients	ADNI/ Local	Developed and employed structure-focused for neurodegeneration CNN architecture (SNeurodCNN)	Analyzed features such as focal structural atrophy and segmented brain structures captured through MRI scans	Nested Cross-Validation	97.8% for para-sagittal viewpoint and 98.1% for mild sagittal viewpoint	[52]
Layer based CNN	To diagnose AD by identifying distinct brain features	Healthy controls (HC) = 897 and Alzheimer's disease (AD) = 1023 patients	ADNI	Devloped and employed eight-layered CNN	Effectively distinguished brain regions of HC and AD subjects	K-Fold Cross-Validation	97%	[53]
Layer based CNN	Identifying discriminative brain regions to classify MCI and AD pathology	Healthy controls (HC) = 1034, Mild cognitive impairment (MCI) = 1149 and Alzheimer's disease (AD) = 1389 patients	AFNI	Integrated eight-layered CNNs with ensemble learning techniques	Identified discriminative brain regions of AD and MCI	K-Fold Cross-Validation	98%	[54]
Layer based CNN	To diagnose AD by identifying distinct brain features	Healthy controls (HC) = 984 and Alzheimer's disease (AD) = 893 patients	ADNI	Employed complex 12-layer CNN model	Classified AD and HC based on distinct brain regions	Nested Cross-Validation	99%	[55]
1D based CNN	To diagnose AD by identifying distinct brain features	Healthy controls (HC) = 1045 and Alzheimer's disease (AD) = 1135 patients	ADNI	Employed one-dimensional CNNs in combination with feedforward neural networks (FFNN)	Efficiently performed clinical diagnoses of AD	K-Fold Cross-Validation	88% for the CNN model and 87% for (FFNN)	[56]
2D and 3D based CNN	To understand the local structural variations within the MRI data and identify the most discriminative features for AD classification	Healthy controls (HC) = 1984 and Alzheimer's disease (AD) = 1048 patients	ADNI/ Local	Employed 2D and 3D CNNs	Obtained enhanced accuracy of AD diagnosis	K-Fold Cross-Validation	90.2% on the ADNI-2 dataset and 85.2% on the ADNI-1 dataset	[59]
2D and 3D based CNN	To capture global subject-level structural changes, facilitating a comprehensive understanding of the MRI data	Healthy controls (HC) = 1049, Mild cognitive impairment (MCI) = 1324 and Alzheimer's disease (AD) = 1297 patients	Local	Integrated multi-view-slice attention with a 2D and 3D CNN	Obtained enhanced accuracy of AD diagnosis	K-Fold Cross-Validation	91.1% for AD and 80.1% for MCI	[57]
2D and 3D based CNN	To perform AD classification by voxel-based decision-making using MRI scans	Healthy controls (HC) = 1193 and Alzheimer’s disease (AD) = 1297 patients	ADNI	Integrating 2D and 3D CNNs and recurrent neural networks (RNNs),	Obtained enhanced accuracy of AD classification	Stratified K-Fold Cross-Validation	96.88%	[58]
Deep 3D based CNNs	To effectively learn multi-level features from brain MRI images for AD classification	Healthy controls (HC) = 1948 and Alzheimer’s disease (AD) = 1398 patients	ADNI	Employed deep 3D CNN to better reflect the complex anatomical structures associated with AD	Obtained enhanced feature selection and classification efficacy	K-Fold Cross-Validation	97.35%	[60]
Deep 3D based CNNs	To accurately differentiate between AD, MCI, and healthy subjects using MRI images	Healthy controls (HC) = 2054, Mild cognitive impairment (MCI) = 1234 and Alzheimer’s disease (AD) = 1198 patients	ADNI/ Local	Employed deep 3D CNN to construct a reference model based on the volumes and thicknesses of brain regions implicated in disease progression	Enhanced distinguishing of AD and MCI diagnosis	Stratified K-Fold Cross-Validation	89%	[61]
Deep 3D based CNNs	To analyze MRI data, single nucleotide polymorphisms (SNPs), and clinical assessments for classification of patients into distinct categories	Healthy controls (HC) = 1294, Mild cognitive impairment (MCI) = 1395 and Alzheimer’s disease (AD) = 1298 patients	ADNI	Employed stacked denoising autoencoders to extract relevant features from clinical and genetic data, subsequently applying a 3D-CNN for imaging analysis	Enhanced distinguishing of AD and MCI diagnosis	Stratified K-Fold Cross-Validation	87%	[62]
Deep 3D based CNNs	To categorizing AD using MRI data	Healthy controls (HC) = 580 and Alzheimer’s disease (AD) = 171 patients	ADNI	Employed two distinct CNN models, each featuring different filter sizes and pooling layers, which are subsequently concatenated at the classification layer	Enhanced distinguishing of AD and MCI diagnosis by effectively capturing and distinguishing relevant features from MRI images	K-Fold Cross-Validation	99.43%	[63]
Deep 3D based CNNs	To categorizing AD using MRI data	Healthy controls (HC) = 490, Mild cognitive impairment (MCI) = 430 and Alzheimer’s disease (AD) = 198 patients	ADNI, OASIS	Employed a deep 3D capsule network along with a pre-trained 3D autoencoder	Enhanced distinguishing of AD and MCI diagnosis by effectively capturing and distinguish relevant features from MRI images	K-Fold Cross-Validation	97.6% accuracy for classifying Alzheimer’s disease against healthy controls (AD/HC) and 94.6% accuracy in distinguishing between mild cognitive impairment and Alzheimer’s disease (MCI/AD)	[46]
3D Jacobian domain based CNN	To diagnose and distinguish AD subjects from HC	Healthy controls (HC) = 1132 and Alzheimer's disease (AD) = 1098 patients	ADNI	Employed 3D Jacobian domain CNN (JD-CNN)	Impressive classification performance for AD without the need for LM detection framework	Stratified K-Fold Cross-Validation	87.4%	[64]

**Abbreviations:** T1-weighted imaging (T1WI), fluorodeoxyglucose positron emission tomography (FDG-PET), Alzheimer’s disease (AD), healthy controls (HC), mild cognitive impairment (MCI), structure-focused for neurodegeneration CNN architecture (SNeurodCNN), feedforward neural networks (FFNN), recurrent neural networks (RNNs), single nucleotide polymorphisms (SNPs), Jacobian domain CNN (JD-CNN), Open access series of imaging studies (OASIS), Alzheimer's Disease Neuroimaging Initiative (ADNI).

**Table 2:** Summary of all studies diagnosing AD by MRI using NC-ANNs.

**Study Types**	**Aim of Study**	**Participants**	**Dataset**	**Strategy**	**Outcomes**	**Validation Approach**	**Performance (Accuracy %)**	**References**
Bi-directional Long Short-Term Memory (BiLSTM) based NC-ANNs	To automate Alzheimer's disease (AD) diagnosis	Healthy controls (HC) = 3489 and Alzheimer's disease (AD) = 5879 patients	Alzheimer's Disease Neuroimaging Initiative (ADNI)	Employed improved adaptive wiener filtering (IAWF) to enhance image quality and normalized global image descriptor (PCA-NGIST) to extract images	Effectively distinguished AD from HC	Stratified K-Fold Cross-Validation	99.22%	[71]
Deep Boltzmann machine based NC-ANNs	To diagnose and classify AD compared to healthy controls and the prediction of progression from MCI to AD	Healthy controls (HC) = 873, Mild cognitive impairment (MCI) = 678 and Alzheimer's disease (AD) = 498 patients	Local	Employed high level latent and shared representation from neuroimaging modalities using deep learning	Effectively distinguished AD from MCI and HC	K-Fold Cross-Validation	95% for distinguishing AD patients from HC individuals and 76% for predicting the conversion of MCI to AD	[73]
Resting-state functional MRI (rs-fMRI) connectivity data based NC-ANN	To distinguish individuals with AD from healthy controls (HC)	Healthy controls (HC) = 987 and Alzheimer's disease (AD) = 798 patients	ADNI/Local	Employed functional connectivity patterns observed in rs-fMRI data using machine learning techniques to improve diagnostic accuracy	Obtained an enhanced differentiation between AD and HC by intrinsic brain activity of subjects in a resting state	K-Fold Cross-Validation	86%	[74]
Resting-state functional MRI (rs-fMRI) connectivity data based NC-ANN	To classify the AD patients and age-and gender-matched control subjects	Healthy controls (HC) = 1098 and Alzheimer's disease (AD) = 1139 patients	ADNI	Employed coupled sophisticated multivariate pattern analysis feature selection algorithm	Effectively achieved near-perfect classification performance	K-Fold Cross-Validation	86.8%	[75]
Salient skeletal model based NC-ANN	To classify AD with high performance	Healthy controls (HC) = 483 and Alzheimer's disease (AD) = 983 patients	Open access series of imaging studies (OASIS)	Developed and employed a salient skeletal model to extract features from MRI scans and identify known brain networks	Skeletal model was found effective in capturing the complex and subtle changes in brain structure that are indicative of AD	Nested Cross-Validation	95%	[76]
Support vector machines (SVM) based NC-ANN	To explore MRI markers aimed at predicting Aβ-positivity in AD pathology	Healthy controls (HC) = 398 and Alzheimer's disease (AD) = 487 patients	ADNI/ OASIS	Employed machine learning (ML) and support vector machines (SVM) to analyze MRI data from 139 patients who underwent brain MRI and amyloid PET-CT scans	Aβ (+) individuals exhibited higher Fazekas scale scores for white matter hyper-intensity (WMH) and cerebral microbleeds (CMB), and identified subtle neuroimaging markers associated with AD pathology	K-Fold Cross-Validation	81.1%	[72]
Support vector machines (SVM) based NC-ANN	To classify AD with high performance	Healthy controls (HC) = 239 and Alzheimer's disease (AD) = 398 patients	Local	Employed stacked network that utilized particle swarm optimization, incorporating both NC-ANN and Support Vector Machine (SVM) algorithms	Effectively distinguished and classified Alzheimer’s disease (AD) from healthy controls (HC)	Nested Cross-Validation	89%	[77]

**Abbreviations:** Alzheimer's disease (AD), Healthy controls (HC), improved adaptive wiener filtering (IAWF), Normalised global image descriptor (NGIST), Mild cognitive impairment (MCI), Resting-state functional MRI (rs-fMRI), Alzheimer's disease neuroimaging initiative (ADNI), machine learning (ML), support vector machines (SVM), cerebral microbleeds (CMB), Open access series of imaging studies (OASIS).

## LIST OF ABBREVIATIONS

AD: Alzheimer's Disease
ADNI: Alzheimer's Disease Neuroimaging Initiative
AI: Artificial Intelligence
AQP4: Aquaporin-4
AUC: Area Under the Curve
Aβ: Amyloid-beta
BiLSTM: Bi-directional Long Short-term Memory
CNNs: Convolutional Neural Networks
CSF: Cerebrospinal Fluid
DBM: Deep Boltzmann Machine
ELM: Extreme Learning Machine
FDG-PET: Fluorodeoxyglucose Positron Emission Tomography
FFNNs: Feedforward Neural Networks
HC: Healthy Controls
IAWF: Improved Adaptive Wiener Filtering
IWHO: Improved Wild Horse Optimization Algorithm
JD-CNN: 3D Jacobian Domain-based Convolutional Neural Network
MCI: Mild Cognitive Impairment
ML: Machine Learning
MRI: Magnetic Resonance Imaging
NACC: National Alzheimer’s Coordinating Center
NC-ANNs: Non-convolutional Artificial Neural Networks
PCA: Principal Component Analysis
PET: Positron Emission Tomography
RNNs: Recurrent Neural Networks
rs-fMRI: Resting-state Functional MRI
SECNN-RF framework: Spectral Enhanced Convolutional Neural Network and Random Forest
SNeurod CNN: Structure-focused Neurodegeneration Convolutional Neural Network
SNPs: Single Nucleotide Polymorphisms
SNUBH: Seoul National University Bundang Hospital
SVM: Support Vector Machine
T1WI: T1-weighted Imaging
XAI: Explainable AI
